# FCKDNet: A Feature Condensation Knowledge Distillation Network for Semantic Segmentation

**DOI:** 10.3390/e25010125

**Published:** 2023-01-07

**Authors:** Wenhao Yuan, Xiaoyan Lu, Rongfen Zhang, Yuhong Liu

**Affiliations:** College of Big Data and Information Engineering, Guizhou University, Guiyang 550025, China

**Keywords:** knowledge distillation, feature condensation, prediction information entropy, feature soft enhancement, semantic segmentation

## Abstract

As a popular research subject in the field of computer vision, knowledge distillation (KD) is widely used in semantic segmentation (SS). However, based on the learning paradigm of the teacher–student model, the poor quality of teacher network feature knowledge still hinders the development of KD technology. In this paper, we investigate the output features of the teacher–student network and propose a feature condensation-based KD network (FCKDNet), which reduces pseudo-knowledge transfer in the teacher–student network. First, combined with the pixel information entropy calculation rule, we design a feature condensation method to separate the foreground feature knowledge from the background noise of the teacher network outputs. Then, the obtained feature condensation matrix is applied to the original outputs of the teacher and student networks to improve the feature representation capability. In addition, after performing feature condensation on the teacher network, we propose a soft enhancement method of features based on spatial and channel dimensions to improve the dependency of pixels in the feature maps. Finally, we divide the outputs of the teacher network into spatial condensation features and channel condensation features and perform distillation loss calculation with the student network separately to assist the student network to converge faster. Extensive experiments on the public datasets Pascal VOC and Cityscapes demonstrate that our proposed method improves the baseline by 3.16% and 2.98% in terms of mAcc, and 2.03% and 2.30% in terms of mIoU, respectively, and has better segmentation performance and robustness than the mainstream methods.

## 1. Introduction

In the field of computer vision, SS tasks occupy a pivotal position [1]. The SS task can classify visual inputs into different semantically interpretable classes. On the micro-level, it is a class resolution of pixels in an image. For example, classifying a pixel point in an image as an airplane or a car, and assigning the same color to the same class of pixel points. Recent work in SS has made tremendous progress, such as deeplabv3+ [2], PSPNet [3], RefineNet [4], and OCRNet [5]. With the development of deep learning and the advent of hardware supporting high-performance computing, deep learning models have become more complex, bringing new challenges to the task of intensive prediction such as SS. First, there is a lack of data available for SS tasks, and it is even more difficult to obtain special data for industrial use. Second, data annotation itself requires a lot of human and material resources, which will greatly limit the development and application of SS in various industries. In addition, from the perspective of development, industrial production is gradually moving towards intelligence. The rise of edge computing indicates that micro-small devices will become the mainstream of the industry, which also requires the models to be lighter and more efficient. In summary, under limited data and cost conditions, the key to the practical application of SS is to move the model to be lightweight and high efficiency.

KD is one of the effective methods to improve the performance of lightweight models, which has been confirmed by many studies. The classical paradigm of the teacher–student model aims to use a deeper or wider network (teacher) to learn more knowledge and to guide and correct another compact network (student). According to the way of knowledge transfer, KD can be categorized as output feature knowledge, intermediate feature knowledge, relational feature knowledge, and structural feature knowledge. The concept of KD was first proposed by Hinton et al. [6] in 2015 and belongs to the way of output feature knowledge. They believed that the outputs contain similar relations between classes. The temperature (T) method was introduced to soften the classification information (soft labels) output by the teacher network and transfer this information as knowledge to the student network to improve performance. Since the output-feature-based KD methods rely on the design of loss functions and are sensitive to parameters, methods combining multiple learning modes have been continuously proposed [7,8]. Benefiting from diverse learning modes such as meta-learning [9], incremental learning [10], and contrast learning [11], output-feature-based KD is applied to cross-domain [12], cross-modal tasks [13], and even special scenarios such as self-distillation [14] and self-supervision [15]. This paves the way for output-feature-based KD to go deeper into various learning tasks. At the same time, the intermediate-feature-based KD methods have also emerged [16,17], which can obtain more abundant knowledge information from the teacher network and solve the problem of single output knowledge information. Compared with the former two, relational feature knowledge and structural feature knowledge pay more attention to the data sample’s relationship between layers [18] and the relationship within features [19].

However, although the above KD methods perform well in many computer vision tasks, they still have limitations for dense prediction tasks such as SS. The KD approach applied to SS requires the teacher network to output feature mapping with more pixel feature representation. Therefore, in the process of teacher network guidance, in addition to knowledge transfer, how to enhance the feature representation capability of the teacher network output is also the key to improving the performance of the student network.

This paper applies an output-feature-based KD to the SS task. We propose a feature condensation method to extract critical foreground knowledge and reduce background noise in the output feature maps to improve the representation of the output features and the difficulty of fitting the student network to the teacher network. To reduce the weakened feature correlation caused by the feature condensation process, we propose a feature soft enhancement method to further intensify the feature representation capability of the teacher network output by improving the elemental correlation of salient regions from the spatial and channel domains. Specific contributions are as follows.

1. According to the calculation rule of pixel information entropy, we designed feature condensation to separate the foreground feature knowledge and background noise of the teacher network outputs. With the help of feature condensation, the teacher network filters the pseudo-knowledge and transfers more accurate foreground feature knowledge to the student network.

2. After feature condensation, we design a feature soft enhancement method, which utilizes the softmax function to calculate the pixel information distribution from the spatial and channel dimensions and converts it into a weight matrix to act on the original feature map, thus enhancing the pixel dependency.

3. Finally, multi-path KD is performed according to the network structure. Extensive experiments using the public Pascal VOC and Cityscapes datasets demonstrate that our proposed method has better performance and robustness than the current mainstream methods. Additionally, the generalization ability test on the fundus retinal vessel segmentation task demonstrates that our method has good cross-domain generalization performance.

The rest of this paper is organized as follows: Section 2 presents the related work. Section 3 introduces the architecture of the proposed FCKDNet, the building blocks of the model, and the objective function. Section 4 presents the experiment settings, qualitative, and quantitative results. Finally, Section 5 summarizes this work.

## 2. Related Work

### 2.1. Knowledge Distillation for Semantic Segmentation

In recent studies, KD-based methods have been widely used to improve the accuracy of lightweight models in SS tasks. Due to different SS scenes and model defects, various pertinent KD methods are proposed. Liu et al. [20] considered dense prediction as a structured prediction and proposed a structured KD scheme (SKD). A pair-wise Markov random field framework is used to improve the continuity of spatial labeling, aligning the pair-wise features learned by the teacher and student networks, thus enabling the student network to learn more structural information. Then, without restricting to pair-wise and pixel-wise dimensions, the outputs of the teacher and student networks are supervised using adversarial training, so that the student network can be approximated in a higher dimension. Different from the study of dense pair-wise relations, Wang et al. [21] focused on the intra-class feature and proposed an intra-class feature variation KD method (IFVD). The features on each pixel are compared to the similarity with various feature centers to represent the intra-class feature variation. Then the most robust intra-class feature variation representation in the teacher network is used to correct the student network. Shu et al. [22] argued that aligning the activation mapping of teacher and student in the spatial domain may bring redundant information to the teacher network. To this end, they proposed a channel-wise distillation method (CWD) to normalize the activation mapping of each channel to obtain the soft probability mapping. The Kullback–Leibler divergences of the teacher and student networks are then minimized to make the distillation process focus more on the significant regions of the channel. Arnaudo et al. [23] proposed a contrastive regularization distillation and applied it to aerial image processing in combination with incremental learning, and achieved superior segmentation accuracy. Yang et al. [24] proposed a cross-image relational KD (CIRKD) for urban road scene segmentation by analyzing the pixel dependencies in global images. Subsequently, Huang et al. [25] argued that extracting better knowledge from a stronger teacher network is the key to improving student network performance. To this end, they constructed a correlation-based loss to capture the intrinsic inter-class relationships from teachers, using the relationship between teacher and student predictions as a knowledge premise. With the prevalence of Transformer in the visual field, Liu et al. [26] proposed a Transformer-based KD framework (TransKD), which learns and guides student transformers through feature maps and patch embeddings of large-scale teacher transformers. After eliminating the use of large pre-training transformers, the model greatly reduced FLOPs by more than 85.0%. In addition, Yuan et al. [27] proposed a novel mutual KD semi-supervised learning framework in combination with consistency regularization. In this framework, multiple teacher networks are used to generate high-quality pseudo-labels to supervise the student network, and a bridge of mutual KD is constructed between teacher networks to achieve multi-channel knowledge sharing.

Contrary to the above methods, our work is more concerned with the quality of the output features of the teacher network. The knowledge value contained in a pixel is evaluated by calculating the information entropy of each pixel. Then a threshold is set to filter pixels with low knowledge values and only pixels with high knowledge values are retained for KD operation. Such an approach can well suppress the influence of useless pseudo-knowledge from the original features transferred to the student network.

### 2.2. Feature Enhancement

Feature enhancement is an important tool to improve model performance. Unlike data augmentation, feature enhancement is to perform pixel-level processing on the middle layer feature maps or output feature maps, and improves the representation ability of image features by improving the dependence between pixels. Hou et al. [28] proposed a coordinate attention ( CA ) module which extracts information from both spatial and channel dimensions, emphasizes useful features while suppressing useless features, and enhances the expression ability of features. To better extract detailed spatial information, Deng et al. [29] proposed a two-stage feature-enhanced attention network (FEANet) to mine multi-level features from both spatial and channel dimensions. Benefiting from the proposed feature enhanced attention module (FEAM), FEANet can retain more spatial information to fuse high-resolution features of RGB-T images and refine segmentation boundary. Ji et al. [30] designed a local-to-global context-aware feature enhancement network (LGCNet) for salient object detection using global context-aware information from foreground/background cues and global feature representations. Zhou et al. [31] proposed a compositional multi-scale feature-enhanced learning approach (COMAL) to improve crowd-counting accuracy. The design of COMAL is accomplished in terms of semantic embedding, crowd feature diversity, and multiscale contextual information. To address the problem of small data samples that make it difficult to achieve robust models, Chen et al. [32] designed a novel attention mechanism on the architecture of meta-learning to highlight class-specific features while weakening background noise. Li et al. [33] proposed a feature-enhancement- and channel-attention-guided single-shot detector (FCSSD). This method performs well in multi-scale object detection and achieves a trade-off in accuracy and inference time.

Different from the design concept of these networks, our method is proposed to further improve the fitting degree of the teacher–student network in the KD process. According to the research of Mirzadeh et al. [34], it is not that the higher the performance of the teacher network is, the more helpful the learning of the student network is. When the gap between the teacher and student networks is too large, it leads to the phenomenon that the student network is more difficult to fit the teacher network. After experimental exploration, enhancing the correlation between feature elements can improve the above problems. To this end, we propose a feature enhancement method based on space and channel to further improve the effectiveness of KD.

## 3. Methodology

### 3.1. Overall Architecture

**Figure 1:**
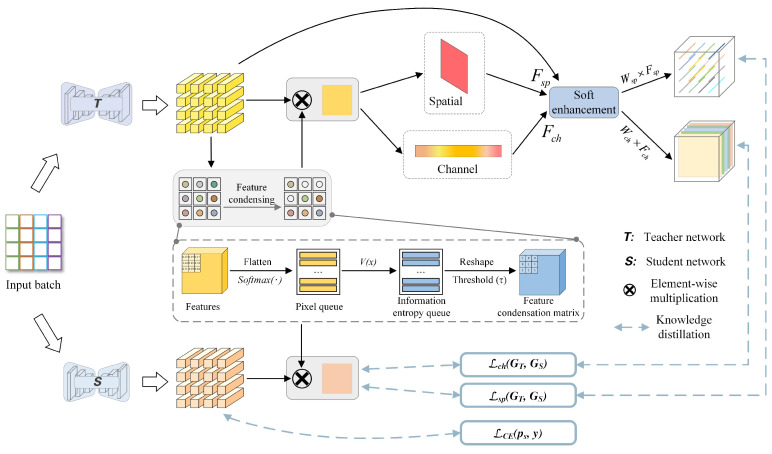
The overall architecture of FCKDNet. In the process of feature soft enhancement based on spatial and channel, we first use maximum pooling and average pooling to obtain salient features in the spatial and channel domains, and weighted multiplication into the original feature mapping to obtain the enhanced feature mapping $F_{sp}$ and $F_{ch}$. Then the softmax function is used for normalization to obtain the soft probability mapping, which is represented by matrices $W_{sp}$ and $W_{ch}$. At this point, $W_{sp}$ and $W_{ch}$ have robust feature probability distribution representation. Finally, the soft probability mappings $W_{sp}$ and $W_{ch}$ are pixel-wise multiplied with the enhanced feature mappings $F_{sp}$ and $F_{ch}$ to complete the entire feature soft enhancement process.

In this paper, we propose a feature condensation KD (FCKDNet) based on the teacher–student model. As shown in Figure 1, the overall architecture consists of the teacher network, the student network, feature condensation operation, space and channel soft enhancement, and KD loss. For both teacher and student networks, we use the classical encoder–decoder structure to extract image features and output pixel class probability distribution feature maps. KD typically requires an excellent network as a teacher. To extract more detailed image features, it is necessary to use a deeper or wider network as the teacher.

Specifically, we feed images into both teacher and student networks by batch for regularization training and obtain the respective output feature maps. To reduce the impact of background noise on foreground pixel prediction, we propose a feature condensation method to separate the foreground pixels from the background pixels in the feature maps output by the teacher network. In the separation phase, the information quantity of each pixel is calculated using the calculation rule of pixel information entropy and the threshold is set to filter the foreground pixels with high information quantity. Then the feature condensation matrix is obtained to multiply with the original feature maps of the teacher and student networks, respectively. The feature maps containing only foreground feature knowledge are obtained. After that, the proposed feature soft enhancement method is used to intensify the foreground features from the spatial and channel dimensions. In the distillation phase, we calculate the similar error between the spatial and channel soft enhancement features of the teacher network and the outputs of the student network as the distillation loss. While the original outputs of the student network calculate the cross-entropy loss with the ground truth and then back-propagating together.

### 3.2. Feature Condensation

Feature condensation is a process of filtering useless information. In this paper, we separate the foreground feature knowledge from the background noise in the feature maps and select only the real and rich foreground feature knowledge for distillation. It will reduce the unnecessary effect of background noise on the student network during distillation. Before that, we need to clarify two issues: (1) how to evaluate the usefulness of a feature element and (2) how to filter out useless information from the output feature map of the teacher network. According to [35], the essence of the output-feature-based KD is to calculate the similarity error at the pixel level for the output feature maps of the teacher and student networks, and thus the variation of element value in the feature maps is crucial. In other words, we can convert the element value into prediction probability, and use the prediction probability to determine the importance of that pixel point. Further, the prediction information entropy can represent the knowledge contribution of each pixel point. Therefore, given a pixel point with a predicted value $\left(x,p_{T}\left(x\right)\right)$, we can calculate the prediction information entropy of that point as:

(1) $$V\left(x\right)=-\sum p_{T}\left(x\right)\log p_{T}\left(x\right)$$

After obtaining the prediction information entropy of pixel points, we can filter out the pixel points with high knowledge contribution by setting a threshold and retaining the position index of these points. Finally, the position index is used to find the pixel value of this position from the original feature maps and retain it as knowledge.

**Figure 2:**
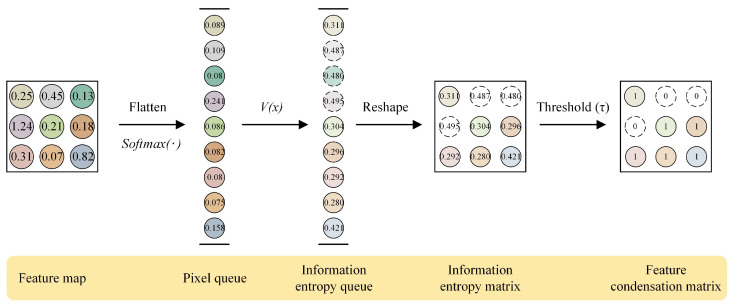
Example of calculating feature condensation matrix.

For the output feature map $F_{T}\in R^{C\times H\times W}$ of the teacher network, where *c* denotes the number of output channels and *H* and *W* denote the spatial dimensions, firstly, we flatten it into the two-dimensional form of $\left(C,HW\right)$ and perform the softmax operation to distribute the pixel prediction values in the range of $\left(0,1\right)$, obtain the pixel queue $Q_{T}$. Then, according to Equation (1), we calculate the prediction information entropy $Q_{T}$ for each pixel in $V\left(Q_{T}\left(x\right)\right)$ to obtain the prediction information entropy queue $Q_{IE}$. After that, we recover $Q_{IE}$ again into a matrix $\left(C,H,W\right)$ of the shape $M_{IE}$ and set a threshold τ to filter the prediction information entropy in $M_{IE}$. Mathematically, the evaluation of the pixel points can be expressed as:

(2) $$V_{E}\left(x\right)=\left\{\begin{array}{c} 1,\;\mathrm{if}\;M_{IE}\left(x\right)\leq \tau \\ 0,\;\mathrm{if}\;M_{IE}\left(x\right)>\tau \end{array}\right.$$

where *x* represents the index in the matrix, and $V_{E}$ represents the feature condensation matrix of the teacher network. Because the prediction information entropy of the pixel point is inversely proportional to its prediction value, that is, the greater the prediction value, the smaller the prediction information entropy. Therefore, we set the pixels whose prediction information entropy is less than or equal to the threshold τ to 1 for retention, and the pixels greater than the threshold τ to 0 for elimination, and then obtain the feature condensation matrix $V_{E}$. Finally, $V_{E}$ is multiplied with the original feature maps of the teacher and student networks and can be expressed as:

(3) $$F_{T-FC}=F_{T}\times V_{E}$$

(4) $$F_{S-FC}=F_{S}\times V_{E}$$

where $F_{T-FC}$ represents the foreground feature matrix of teacher network output, $F_{S-FC}$ represents the foreground feature matrix of student network output, $F_{S}$ represents the original feature map of student network, and the notation × represents element-wise multiplication in the matrix. The calculation of $V_{E}$ is shown in Figure 2.

### 3.3. Feature Soft Enhancement

In order to intensify the representation ability of the teacher network after obtaining the foreground feature map $F_{T-FC}$, inspired by reference [36], we design a feature soft enhancement method based on spatial and channel dimensions to improve pixel affinity in $F_{T-FC}$. As shown in Figure 1, the max pooling and average pooling firstly are used to extract significant feature elements from the spatial and channel dimensions of $F_{T-FC}$, and then we weighted multiply them to the original foreground feature map $F_{T-FC}$ to obtain $F_{sp}$ and $F_{ch}$, respectively. Then the softmax function is used to calculate the probability distribution of the dependence between pixels. The larger the calculation value, the stronger the relative dependence. The probability distribution matrix $W_{sp}$ of $F_{sp}$ can be expressed as:

(5) $$W_{sp}=\mathrm{softmax}(\frac{e^{x_{i}}}{\sum_{i=1}^{HW}e^{x_{i}}})$$

where *x* is the element value corresponding to pixel index *i* in $F_{sp}$, and HW is the length of feature matrix. Similarly, the probability distribution matrix $W_{ch}$ of $F_{ch}$ can be obtained. Finally, we multiply the probability distribution matrices $W_{sp}$ and $W_{ch}$ of spatial and channel dimensions with $F_{sp}$ and $F_{ch}$, respectively, to realize feature soft enhancement. The calculation formulas of the spatial soft enhancement feature $F_{sp}^{{}^{\prime }}$ and the channel soft enhancement feature $F_{ch}^{{}^{\prime }}$ are:

(6) $$F_{sp}^{{}^{\prime }}=F_{sp}\times W_{sp}$$

(7) $$F_{ch}^{{}^{\prime }}=F_{ch}\times W_{ch}$$

### 3.4. Design of Knowledge Distillation

For the KD phase, we use a distillation method similar to similarity-preserving KD [16]. This method obtains its own similarity matrix by calculating the inner product of the corresponding output feature maps of the teacher and student networks, respectively. Then, the mean square error (MSE) is used to measure the two similarity matrices such that the teacher and student networks produce similar activations for the same classes, thus retaining knowledge similar to the teacher network in the student network’s feature maps. The output of the teacher network is given as the spatial soft enhancement feature $F_{sp}^{{}^{\prime }}$ and the channel soft enhancement feature $F_{ch}^{{}^{\prime }}$, and the output of the student network is given as the foreground feature matrix $F_{S-FC}$. The similarity matrix can be expressed as:

(8) $${\tilde{G}}_{sp}=F_{sp}^{{}^{\prime }}\cdot F{}_{sp}^{{}^{\prime }T}\;;\;G_{sp[i,:]}={\tilde{G}}_{sp\left[i,:\right]}/{∥{\tilde{G}}_{sp\left[i,:\right]}∥}_{2}$$

(9) $${\tilde{G}}_{ch}=F_{ch}^{{}^{\prime }}\cdot F{}_{ch}^{{}^{\prime }T}\;;\;G_{ch[i,:]}={\tilde{G}}_{ch\left[i,:\right]}/{∥{\tilde{G}}_{ch\left[i,:\right]}∥}_{2}$$

(10) $${\tilde{G}}_{S-FC}=F_{S-FC}\cdot F{}_{S-FC}^{T}\;;\;G_{S-FC[i,:]}={\tilde{G}}_{S-FC\left[i,:\right]}/{∥{\tilde{G}}_{S-FC\left[i,:\right]}∥}_{2}$$

where we use the row-wise L2 normalization to obtain the similarity matrices $G_{sp}$, $G_{ch}$, and $G_{S-FC}$, and the notation [i,:] denotes the *i*th row in the matrix. Then, the spatial and channel-based KD loss can be defined as:

(11) $$L_{sp}\left(G_{T},G_{S}\right)=\frac{1}{b^{2}}\sum_{i\in I}{∥G_{sp}^{i}-G_{S-FC}^{i}∥}_{F}^{2}$$

(12) $$L_{ch}\left(G_{T},G_{S}\right)=\frac{1}{b^{2}}\sum_{i\in I}{∥G_{ch}^{i}-G_{S-FC}^{i}∥}_{F}^{2}$$

where *b* represents the number of batches in the feature map, ${∥\cdot ∥}_{F}$ represents the Frobenius norm, and *i* represents the *i*th element in the matrix. *i* can index all elements in the teacher–student matrix and calculate the mean element-wise squared difference. Finally, we define the total loss function as:

(13) $$L=\left(1-\gamma \right)L_{CE}\left(p_{s},y\right)+\frac{\gamma }{2}L_{sp}\left(G_{T},G_{S}\right)+\frac{\gamma }{2}L_{ch}\left(G_{T},G_{S}\right)$$

where $L_{CE}\left(\cdot \right)$ represents the cross-entropy loss, $p_{s}$ is the original prediction of the student network, *y* is the ground truth, and γ is the loss balancing hyperparameter.

## 4. Experiments

### 4.1. Experimental Setup

**Dataset.** We perform all experiments on three public datasets with different application scenes. (1) Pascal VOC [37] is a visual image dataset available for object detection and SS. It has 20 classes with one background class containing 1464 images for training, 1449 images for validation, and 1456 images for testing. (2) Cityscapes [38] is a dataset of urban road scenes with 19 classes, containing 2975 images for training, 500 images for validation, and 1525 images for testing. (3) DRIVE [39] is a fundus retinal vessel segmentation dataset that is used to test the cross-domain generalization performance of the proposed method. It contains 40 images with a resolution of 584×565, where 20 images are used for training and 20 images for testing.

**Evaluation metrics.** We employ mean intersection over union (mIoU) and mean accuracy (mAcc) to measure the segmentation performance. In the cross-domain generalization performance experiment, we add sensitivity (Sen), specificity (Spe), and the Dice similarity coefficient (DSC) as metrics for medical image segmentation. They are defined as:

(14) $$\mathrm{mIoU}=\frac{1}{K}\frac{TP}{FP+TP+FN}$$

(15) $$\mathrm{mAcc}=\frac{1}{K}\frac{TP+TN}{TP+FP+TN+FN}$$

(16) $$\mathrm{Sen}=\frac{TP}{TP+FN}$$

(17) $$\mathrm{Spe}=\frac{TN}{TN+FP}$$

(18) $$\mathrm{DSC}=\frac{2TP}{FP+2TP+FN}$$

where *TP* denotes the number of pixels of the target object correctly divided into the target region; *TN* denotes the number of pixels of the background part correctly segmented into the background part; *FP* denotes the number of pixels of the background part wrongly segmented into the target region; *FN* denotes the number of pixels of the target object wrongly segmented into the background part; *K* represents the number of classes.

**Network architechtures.** For all experiments, we use DeeplabV3+ [2] as the overall architecture of the SS network. The deep ResNet-101 (Res101) [40] and wide WideResNet-50-2 (WRes50) [41] backbone are used as powerful teacher networks. For the student network, we use the lightweight MobileNetV2 (MBV2) [42] and ResNet-18 (Res18) [40] as the backbone.

**Training details.** Based on the standard data augmentation, we apply random flipping and scaling in the range of [0.5,2]. To fit the input size of the network, the images were cropped to 512×512 for Pascal VOC and DRIVE, and 640×640 for Cityscapes. Throughout the training process, the network was optimized using SGD with an initial learning rate of 0.001 for Pascal VOC and DRIVE and 0.01 for Cityscapes, a momentum of 0.9, and a batch size of 8. The number of total training iterations is 30 K for Pascal VOC and Cityscapes, and 1.2 K for DRIVE. In addition, due to the different complexities of the dataset scenes, we set the feature condensation threshold τ and the loss balancing hyperparameter γ differently. For Pascal VOC and DRIVE, τ is set to 0.45, and γ is set to 0.6; for Cityscapes, τ is set to 0.25, and γ is set to 0.4. All work is done on a 20.04 Ubuntu system and a GeForce RTX3090 GPU.

### 4.2. Comparison with Other Methods

Our proposed method is to optimize the output features of the teacher network. It is necessary to verify the effectiveness of our method using different types of output features. Therefore, we use the deep network (Res101) and wide network (WRes50) as the backbone of the teacher network namely DeepLabV3+-Res101 and DeepLabV3+-WRes50. Additionally, we add the mainstream distillation methods SKD [20], CWD [21], and IFVD [22] to the same network architecture for comparison experiments. To ensure the fairness of the experiments, we complete and analyze all comparison experiments under the same experimental setting and training details.

#### 4.2.1. Results on the Deep Teacher Network

We measure the number of parameters, computational complexity of the teacher and student networks, and the computational time required for each KD method. Experiments were performed using the Pascal VOC and Cityscapes datasets, and the results are shown in Table 1. It can be intuitively seen that there is only a small difference between the performance of the student network and the teacher network with a significant difference in model parameters and FLOPs, and the performance of the student network is improved after embedding the KD. CWD and IFVD perform better in terms of computational time with 15.9 ms and 18.3 ms, respectively. The computational time of our proposed FCKD is 20.6 ms, which is slightly inferior compared to the first two but improves by 3.9 ms compare to SKD. Therefore, our method has a strong competitive advantage in computing costs. In terms of segmentation performance, compared to the state-of-the-art methods, the proposed FCKD has the most significant improvement on the original student network and is closest to the results of the teacher network. Specifically, for the student network DeepLabV3+-MBV2, mAcc improves by 3.16% and mIoU improves by 2.03% on Pascal VOC, mAcc improves by 2.98% and mIoU improves by 2.30% on Cityscapes; for the student network DeepLabV3+-Res18, mAcc improves by 3.23% and mIoU improves by 1.96% on Pascal VOC, mAcc improves by 2.64% and mIoU improves by 1.65% on Cityscapes. Compared to CWD, with the best segmentation performance among mainstream methods, FCKD has a small performance improvement. In particular, FCKD improves mAcc by 0.36% and mIoU by 0.20% on Pascal VOC, and mAcc by 0.34% and mIoU by 0.36% on Cityscapes compare to CWD (calculated from the average of the results of the two student networks). In addition, IFVD based on class-level feature representation is similar to our method, while the difference is that IFVD compares the similarity of features on each pixel with various feature centers, and our method focuses more on ensuring the integrity of the feature regions when feature condensation is performed. From the experimental results, IFVD has a faster computational speed, while our FCKD has better segmentation performance. The performance difference between FCKD and IFVD on DeepLabV3+-MBV2 is more significant. Compared to IFVD, FCKD improves mAcc by 0.86% and mIoU by 0.71% on Pascal VOC, mAcc by 1.01% and mIoU by 1.31% on Cityscapes. In summary, our proposed method has better segmentation performance and algorithmic robustness under multiple data domains.

**Figure 3:**
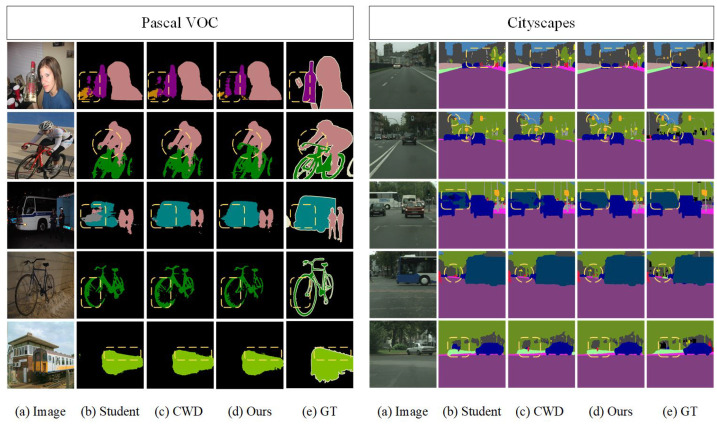
Segmentation results using the deep teacher network and the student network DeepLabV3+-MBV2 on the validation sets of Pascal VOC and Cityscapes: (**a**) raw images, (**b**) the original student network without KD, (**c**) channel-wise distillation, (**d**) our method, and (**e**) ground truth.

**Figure 4:**
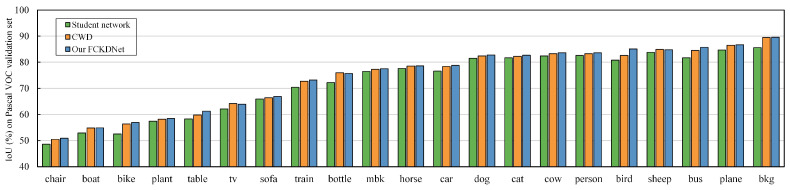
Illustration of individual class IoU scores over the student network DeepLabV3+-MBV2, the mainstream CWD, and our proposed FCKDNet on the Pascal VOC validation set.

To show the segmentation performance difference more visually, we visualize the segmentation results of the student network, CWD, and the proposed FCKDNet, as shown in Figure 3. The segmentation differences are marked using yellow dashed lines. It can be seen that FCKDNet has more accurate segmentation for objects close to the background color due to the reduction of the influence of misclassified pixels in the background during the feature similarity calculation, such as the bottle in the first row of Pascal VOC, the bus in the third row, and the distant street lamp in the second row of Cityscapes. The segmentation performance of the student network is slightly inferior due to its own performance limitations. In addition, Figure 4 shows in more detail the specific IoU scores of individual classes in the Pascal VOC validation set. We can see that our FCKDNet has better segmentation results compared to the student network and CWD. There is a significant elevation of small objects and more regular objects. For example, the segmentation of birds, buses, and tables improves by 4.3%, 4.0%, and 3.2%, respectively, compared to the student network, and improves by 2.5%, 1.2%, and 0.5% compared to CWD.

#### 4.2.2. Results on the Wide Teacher Network

**Figure 5:**
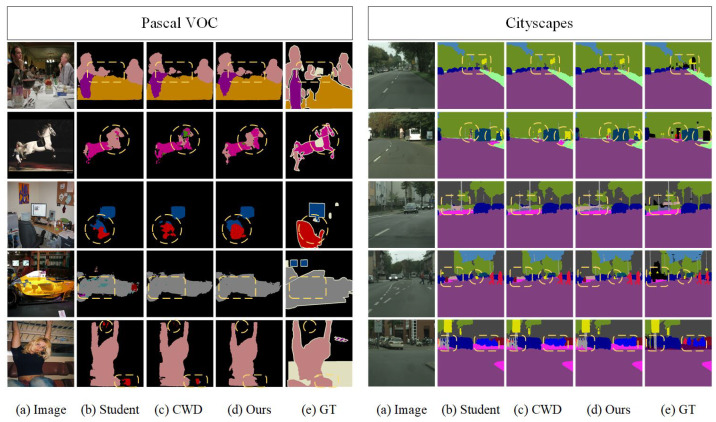
Segmentation results using the wide teacher network and the student network DeepLabV3+-MBV2 on the validation sets of Pascal VOC and Cityscapes: (**a**) raw images, (**b**) the original student network without KD, (**c**) channel-wise distillation, (**d**) our method, and (**e**) ground truth.

The wide teacher network ensures that rich features, e.g., texture features in different orientations and frequencies, are learned at each layer. Then, our proposed feature condensation operation and feature soft enhancement method are used to further highlight feature information from the pixel level. Table 2 shows the experimental results using the wide teacher network. It can be seen that our proposed FCKDNet is closer to the segmentation results of the teacher network compare to other conventional methods. Specifically, for the student network DeepLabV3+-MBV2, our proposed FCKD improves mAcc by 2.43% and mIoU by 1.74% on Pascal VOC, and mAcc by 2.67% and mIoU by 2.12% on Cityscapes. For the student network DeepLabV3+-Res18, mAcc and mIoU improve by 2.26% and 1.65% on Pascal VOC, respectively, and both metrics improve by 1.71% and 1.04% on Cityscapes, respectively. Among the mainstream methods, CWD has the best segmentation performance, IFVD is suboptimal, and SKD has the smallest improvement. Compared to CWD, the proposed FCKD is significantly enhanced on Cityscapes, where mAcc and mIoU improve by 0.34% and 0.36%, respectively. Compared to IFVD, FCKD achieves a better performance on Cityscapes, where mAcc and mIoU improve by 1.01% and 0.86%, respectively. Compared to SKD, FCKD significantly improved the results on Pascal VOC, where mAcc and mIoU improve by 1.28% and 1.19%, respectively (calculated from the average of the results of the two student networks). In summary, the experimental results demonstrate the applicability of our FCKDNet to the wide teacher network as well. Figure 5 shows the qualitative results for the student network, CWD, and our proposed FCKDNet. The areas of significant segmentation differences are marked using yellow dashed lines in the results. We can observe that FCKDNet has the best segmentation results and the fewest misclassified pixel regions.

### 4.3. Ablation Study

In this section, we conduct detailed ablation experiments for each module in the network. We use the student network as a baseline on which we add the teacher network, feature condensation, spatial soft enhancement, and channel soft enhancement successively. All experiments are performed on Pascal VOC. As shown in Table 3, direct use of the teacher network for KD improves baseline by 1.85% on mAcc and 1.14% on mIoU. Using feature condensation on the teacher network improves baseline 2.38% on mAcc and 1.66% on mIoU. Additionally, the baseline improves by 2.78% and 3.04% on mAcc, and 1.78% and 1.85% on mIoU, respectively, using spatial and channel feature soft enhancement. The results lead to two conclusions: (1) feature condensation has a good separation of foreground feature knowledge and background noise, and has a higher performance improvement for the network relative to the spatial soft enhancement and channel soft enhancement; (2) channel soft enhancement captures richer feature information than spatial soft augmentation. Finally, using all modules maximizes the performance with a 3.16% improvement in terms of mAcc and 2.03% improvement in terms of mIoU compared to the baseline. In summary, our proposed method improves the representation ability of the teacher network’s output features and improves the problem that the teacher–student network is difficult to fit during the training period.

**Figure 6:**
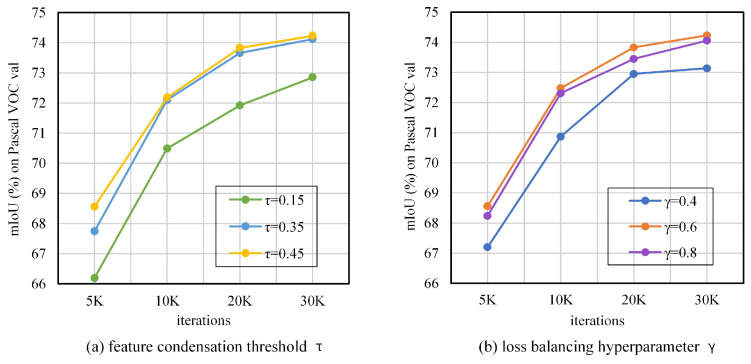
Impact of the feature condensation threshold τ and the loss balancing hyperparameter γ on our proposed FCKDNet.

Adjusting the feature condensation threshold τ and the loss balancing hyperparameter γ is the key to improving the network performance. We set τ to 0.15, 0.35, and 0.45, and γ to 0.4, 0.6, and 0.8 for the experiments. The impact on the network is shown in Figure 6. For τ, when τ = 0.15, the performance improvement is the least, which indicates that some feature pixels are lost when filtering background pixels. When τ = 0.45, the performance improvement is the highest, while the improvement is not significant with respect to τ = 0.35, indicating that 0.45 is closer to the optimal value. For γ, it can be seen that the KD effect is not significant at γ = 0.4; when γ = 0.8, the distillation weight is so high that it ignores the supervision of the network by the ground truth; the network performance is best at γ = 0.6.

### 4.4. Cross-Domain Generalization Ability

Finally, we discuss the cross-domain generalization ability of FCKDNet. Different from the natural-environment-style dataset used in the above section, we use a medical image dataset to illustrate the cross-domain segmentation performance of the proposed method. This is a fundus retinal vessel segmentation task, and since the DRIVE dataset used does not contain a validation set, we divide the 20 images of the training set into 16 for training and 4 for validation. Finally, the allocation ratio of the training set, validation set, and test set for the experiment is 16:4:20. Moreover, we choose the classic segmentation network SegNet [43] and the high-performance U-Net [44] and R2U-Net [45] in the field of medical image segmentation for experimental comparison. In addition to Acc and IoU, we add Sen, Spe, and DSC as evaluation metrics for medical image segmentation. The higher values of Sen, Spe, and DSC, the better performance of the network.

**Figure 7:**
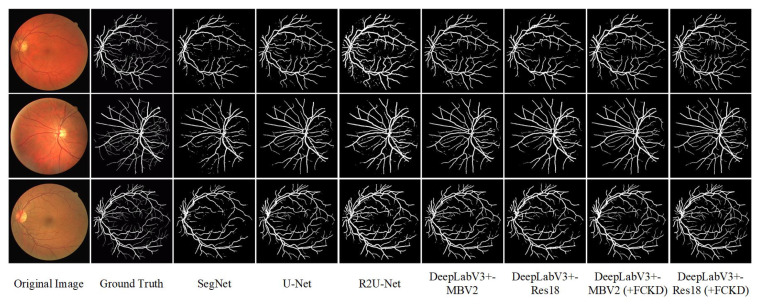
The visual results of fundus vascular segmentation.

The experiments are performed according to the training details in Section 4.1, and the results are shown in Table 4. It can be found that SegNet has the highest score of 78.9% on Sen. DeepLabV3+-MBV2 and DeepLabV3+-Res18, which used FCKD (DeepLabV3+-MBV2 (+FCKD) and DeepLabV3+-Res18 (+FCKD)), are 76.6% and 77.8% on Sen, respectively. Compared with U-Net and R2U-Net, they have a smaller gap with SegNet. In the Spe metric, DeepLabV3+-MBV2 and DeepLabV3+-Res18 have insignificant performance, while DeepLabV3+-MBV2 (+FCKD) and DeepLabV3+-Res18 (+FCKD) improve on them by 0.4% and 0.9%, respectively, which is comparable to U-Net. More, our DeepLabV3+-MBV2 (+FCKD) and DeepLabV3+-Res18 (+FCKD) have superior performance on DSC, Acc, and IoU. DeepLabV3+-Res18 (+FCKD) obtains the highest scores on DSC, Acc, and IoU with 81.8%, 96.5%, and 69.2%, respectively. Additionally, DeepLabV3+-MBV2 (+FCKD) has the most significant improvement over DeepLabV3+-MBV2, improving by 2.2%, 1.3%, and 3.1% on DSC, Acc, and IoU, respectively. Figure 7 shows the qualitative results for each network. It can be seen that DeepLabV3+-MBV2 (+FCKD) and DeepLabV3+-Res18 (+FCKD) have more detailed segmentation performance based on DeepLabV3+-MBV2 and DeepLabV3+-Res18, for example, the segmentation is clearer and more continuous on the fine vessels. Compared with U-Net and R2U-Net, the segmentation results of our method are richer and closer to the ground truth. In general, our FCKDNet is competent for the task of fundus retinal vessel segmentation and achieves better results. In other words, our method has good generalization performance on the cross-domain segmentation task.

## 5. Conclusions

In this paper, a novel feature condensation KD method is proposed for SS. The method is able to separate foreground feature knowledge and background noise at the pixel level in the output features of the teacher network. Then, a feature soft enhancement method based on spatial and channel dimensions is used for the foreground feature knowledge to further improve the feature representation ability of the network. Finally, the enhanced features of the teacher network are used to distill knowledge with the student network. Compared with the current mainstream KD methods, our method can effectively help the teacher network filter pseudo-knowledge and improve student network performance. Experiments on public datasets demonstrate the effectiveness and good cross-domain generalization performance of our FCKDNet. In addition, our method still suffers from shortcomings such as the inability to adaptively find the optimal solution of the threshold during the feature condensation process, which may cause over-separation of effective knowledge. In the future, we will continue to optimize our network, and we hope our work will inspire more researchers to investigate feature filtering and apply it to segmentation KD.

## Figures and Tables

**Table 1:** Performance comparison with mainstream distillation methods using the deep teacher network on Pascal VOC and Cityscapes. We tag the teacher as T and the student as S. FLOPs are measured based on the fixed size of 512×512. The computational time represents the inference time of the teacher and student networks and the computational time of each distillation method. The bold number denotes the best result in each block.

Methods	Params (M)	FLOPs (G)	Computational Time (ms)	Val mAcc (%)		Val mIoU (%)	
				Pascal VOC	Cityscapes	Pascal VOC	Cityscapes
T: DeepLabV3+-Res101	56.0	1870.0	13.7	89.64	87.24	77.67	77.46
S: DeepLabV3+-MBV2	5.0	229.8	5.3	81.35	80.59	72.20	70.44
+SKD [20]			24.5	82.42	81.78	72.86	71.58
+CWD [22]			**15.9**	84.27	83.23	74.05	72.26
+IFVD [21]			18.3	83.65	82.56	73.52	71.43
+FCKD (ours)			20.6	**84.51**	**83.57**	**74.23**	**72.74**
S: DeepLabV3+-Res18	20.3	784.5	4.7	82.69	83.05	73.18	73.21
+SKD [20]			24.5	84.73	84.22	74.35	73.78
+CWD [22]			**15.9**	85.45	85.17	74.92	74.62
+IFVD [21]			18.3	85.14	84.83	74.77	74.15
+FCKD (ours)			20.6	**85.92**	**85.51**	**75.14**	**74.86**

**Table 2:** Performance comparison with mainstream distillation methods using the wide teacher network on Pascal VOC and Cityscapes. We tag the teacher as T and the student as S. FLOPs are measured based on the fixed size of 512×512. The computational time represents the inference time of the teacher and student networks and the computational time of each distillation method. The bold number denotes the best result in each block.

Methods	Params (M)	FLOPs (G)	Computational Time (ms)	Val mAcc (%)		Val mIoU (%)	
				Pascal VOC	Cityscapes	Pascal VOC	Cityscapes
T: DeepLabV3+-WRes50	72.2	2653.9	7.6	86.58	85.45	76.38	75.62
S: DeepLabV3+-MBV2	5.0	229.8	5.3	81.35	80.59	72.20	70.44
+SKD [20]			24.5	82.64	81.57	72.76	71.21
+CWD [22]			**15.9**	83.38	82.74	73.40	72.13
+IFVD [21]			18.3	83.06	82.28	73.15	71.27
+FCKD (ours)			20.6	**83.78**	**83.26**	**73.94**	**72.56**
S: DeepLabV3+-Res18	20.3	784.5	4.7	82.69	83.05	73.18	73.21
+SKD [20]			24.5	83.53	83.42	73.64	73.55
+CWD [22]			**15.9**	84.62	84.46	74.51	74.10
+IFVD [21]			18.3	84.15	83.73	74.16	73.87
+FCKD (ours)			20.6	**84.95**	**84.76**	**74.83**	**74.25**

**Table 3:** Ablation study of our method on the validation set of Pascal VOC. Student (Baseline): we set DeepLabV3+-MBV2 as the student network and baseline. Teacher: we set DeepLabV3+-Res101 as the teacher network. FC: feature condensation. SSE: spatial soft enhancement that proposed. CSE: channel soft enhancement that proposed. The bold number denotes the best result.

Student (Baseline)	Teacher	FC	SSE	CSE	Val mAcc (%)	Val mIoU (%)
*√*	-	-	-	-	81.35	72.20
*√*	*√*	-	-	-	83.20	73.34
*√*	*√*	*√*	-	-	83.73	73.86
*√*	*√*	*√*	*√*	-	84.13	73.98
*√*	*√*	*√*	-	*√*	84.39	74.05
*√*	*√*	*√*	*√*	*√*	**84.51**	**74.23**

**Table 4:** Cross-domain generalization performance study of our method on the DRIVE dataset. The bold number denotes the best result.

Method	Sen (%)	Spe (%)	DSC (%)	Acc (%)	IoU (%)
SegNet [43]	**78.9**	86.7	62.1	85.3	45.0
U-Net [44]	73.6	96.3	79.6	94.8	66.1
R2U-Net [45]	76.4	**97.2**	81.5	96.2	68.8
DeepLabV3+-MBV2	73.3	96.2	79.3	95.1	65.7
DeepLabV3+-Res18	74.1	95.4	80.8	95.3	67.8
DeepLabV3+-MBV2 (+FCKD)	76.6	96.6	81.6	96.4	68.9
DeepLabV3+-Res18 (+FCKD)	77.8	96.3	**81.8**	**96.5**	**69.2**

## Data Availability

Publicly available datasets were used in this study. The Pascal VOC 2012 dataset can be found here: http://host.robots.ox.ac.uk/pascal/VOC/voc2012/index.html (accessed on 1 October 2022). The Cityscapes dataset can be found here: https://www.cityscapes-dataset.com/ (accessed on 1 October 2022). The DRIVE dataset can be found here: https://drive.grand-challenge.org/ (accessed on 1 October 2022).
